# Primary outcomes of the VIDI study: phase 2, double-masked, randomized, active-controlled study of ASP8232 for diabetic macular edema

**DOI:** 10.1186/s40942-019-0178-7

**Published:** 2019-08-01

**Authors:** Quan Dong Nguyen, Yasir J. Sepah, Brian Berger, David Brown, Diana V. Do, Alberto Garcia-Hernandez, Sunil Patel, Firas M. Rahhal, Yevgeniy Shildkrot, Ronny W. Renfurm, the VIDI Research Group

**Affiliations:** 1 Byers Eye Institute, Stanford University School of Medicine, 2370 Watson Court, Suite 200, Palo Alto, CA 94303 USA; 2 Ocular Imaging Research and Reading Center, Sunnyvale, CA USA; 3 Retina Research Center, PLLC, 3705 Medical Parkway, Austin, TX 78705 USA; 4 Retina Consultants of Houston, Houston, TX USA; 5 Formerly With Astellas Pharma Europe BV, Sylviusweg 62, 2333 BE Leiden, The Netherlands; 6 Integrated Clinical Research, LLC, 5441 Health Center Drive, Abilene, TX 79606 USA; 7 Retina Vitreous Associates Medical Group, 9001 Wilshire Boulevard, Suite 301, Beverly Hills, CA 90211 USA; 8 University of Virginia, 1300 Jefferson Park Avenue, Charlottesville, VA 22908 USA

**Keywords:** ASP8232, VAP-1 inhibitor, Center-involved diabetic macular edema, Central subfield thickness, Ranibizumab, Clinical trial

## Abstract

**Background:**

ASP8232 is a potent and specific small molecule vascular adhesion protein-1 (VAP-1) inhibitor. This study evaluated the effect of ASP8232 on excess retinal thickness when given alone or in combination with ranibizumab in patients with center-involved diabetic macular edema (CI-DME).

**Methods:**

This was a phase 2a, placebo and sham-injection controlled, double-masked, randomized, parallel-group clinical trial. Participants were patients with CI-DME and central subfield thickness (CST) ≥ 375 µm in the study eye as assessed by spectral domain optical coherence tomography. Eligible patients were randomized to (1) daily oral ASP8232 40 mg monotherapy; (2) combination therapy of daily oral ASP8232 40 mg and monthly intravitreal ranibizumab 0.3 mg; or (3) monthly intravitreal ranibizumab 0.3 mg monotherapy. The treatment period was 12 weeks. CST and best corrected visual acuity (BCVA) were assessed at baseline and at Weeks 2, 4, 8, 12, 16 and 24. The primary outcome was the mean percent change from baseline in excess CST at Week 12. Secondary outcomes were BCVA, safety and tolerability, and pharmacokinetic and pharmacodynamic characteristics of ASP8232.

**Results:**

After 12 weeks, the mean (95% confidence interval) percent change in excess CST was 11.4% (− 15.0%, 37.8%) in the ASP8232 group, − 61.7% (− 86.1%, − 37.2%) in the ASP8232/ranibizumab group, and − 75.3% (− 94.8%, − 55.8%) in the ranibizumab group. The change from baseline in the two ranibizumab arms was statistically significant (*P *< 0.001) as was the difference between the ranibizumab groups and the ASP8232 group (*P *< 0.001). Mean (SD) increase in BCVA score from baseline was 3.1 (7.3) in the ASP8232 group, 5.2 (7.1) in the ASP8232/ranibizumab group, and 8.2 (9.5) in the ranibizumab group. The increase from baseline in BCVA score was statistically and clinically significant in the ranibizumab group compared with the ASP8232 group (*P* = 0.015). ASP8232 resulted in near complete inhibition of plasma VAP-1 activity whilst ranibizumab had no effect.

**Conclusions:**

Near complete inhibition of plasma VAP-1 activity with ASP8232 had no effect on CST in patients with CI-DME. Furthermore, combination therapy did not provide additional benefit to treatment with ranibizumab alone, which significantly reduced CST and improved BCVA.

*Trial registration* clinicaltrials.gov; NCT02302079. Registered on November 26, 2014

**Electronic supplementary material:**

The online version of this article (10.1186/s40942-019-0178-7) contains supplementary material, which is available to authorized users.

## Background

Diabetic retinopathy (DR) is a common complication of diabetes mellitus that leads to loss of vision and blindness among working age adults [1–3]. During progression of DR, patients can develop diabetic macular edema (DME), which is characterized by the thickening of the macula caused by the breakdown of the blood-retinal barrier and consequent retinal vascular hyperpermeability [3]. In 2010, the global prevalence of DR among adults with diabetes mellitus aged 20–79 years was estimated to be 34.6% for any DR and 6.81% for DME [4]. DME is the leading cause of vision loss among patients with DR. It is associated with the type of diabetes, and increases with the duration and severity of disease [5, 6]. Other significant risk factors common to DR and DME include hyperglycemia and hypertension [4]. DME negatively impacts patients’ health-related quality of life and represents an economic burden due to the increased use of healthcare resources by affected patients [7, 8].

While there is no curative treatment available for DME, laser photocoagulation represents an effective treatment to preserve vision. However, this treatment modality is limited by its inability to restore vision once it has been lost [9]. The current standard of care for DME includes intravitreal anti-vascular endothelial growth factor (VEGF) antibodies and corticosteroids [9]. Clinical studies have confirmed that monthly intravitreal treatment with the anti-VEGF antibody ranibizumab can improve vision, with up to 45% of patients gaining ≥ 15 letters in best-corrected visual acuity (BCVA) after 24 months [10, 11]. Similar improvements were found after treatment with the anti-VEGF antibodies aflibercept [12–14] and with bevacizumab, an approved treatment for colon cancer that is commonly used off label for DME [9, 15]. Despite the proven efficacy of VEGF inhibitors, the requirement of frequent injections causes a high rate of treatment discontinuation among patients with DME and represents a major limitation [16]. Moreover, the presence of potential side effects and the significant proportion of patients who do not respond to treatment [10, 12] suggest that there remains a need for the development of improved therapies for DME.

Vascular adhesion protein-1 (VAP-1) belongs to the family of copper-containing amine oxidase/semicarbazide-sensitive amine oxidases that catalyze the oxidative deamination of primary amines with subsequent production of aldehyde, ammonium, and hydrogen peroxide, which are involved in oxidative stress and are cytotoxic. VAP-1 is expressed in vascular endothelium (including renal and retinal capillaries), smooth muscle cells, hepatic stromal cells and adipocytes [17, 18]. The enzymatic action stimulates leukocyte trafficking to the interstitium and therefore has a pro-inflammatory action [17, 18]. After inflammatory stimulus, a soluble form of VAP-1, which retains its enzymatic activity, is released into circulation from the endothelial cells. Elevated levels of soluble VAP-1 have been found in the serum of diabetic patients [19] and in the vitreous fluid of patients with proliferative DR (PDR) compared with non-PDR patients [20, 21]. Recently, it was shown that VEGF induces soluble VAP-1 release in retinal capillaries and thereby induces increased production of reactive oxygen species [22]. In experimental models of uveitis and DR in rats, VAP-1 was suggested to play a significant role in leucocyte recruitment into retinal endothelium [23–25]. Such evidence suggests that inhibition of VAP-1 may represent a novel therapeutic strategy for DR and DME.

ASP8232 is a potent and specific small molecule VAP-1 inhibitor. In a streptozocin-induced rat model of DME, ASP8232 inhibited plasma VAP-1 activity and improved retinal hyperpermeability and plasma total antioxidant status. In combination with intravitreal anti-rat VEGF antibody, ASP8232 was more effective in reducing ocular hyperpermeability compared with either the anti-ratVEGF antibody or ASP8232 alone (data on file).

In multiple pharmacology and safety/toxicity experimental studies, ASP8232 had low acute toxicity and showed no effect on central nervous system, cardiovascular, and respiratory functions or fertility and early embryonic development, and had no genotoxic or teratogenic potential. ASP8232 was tested in two phase 1 studies in healthy subjects, patients with renal impairment, and patients with type 2 diabetes mellitus. ASP8232 was administered in doses up to 6000 mg as single dose and up to 800 mg as multiple doses in healthy subjects. In subjects with renal impairment and diabetes, ASP8232 was administered in a dose of 150 mg for 4 weeks (unpublished data from first-in-man study, NCT02218099). ASP8232 was safe and well-tolerated, and pharmacokinetic and pharmacodynamic modelling and simulations suggested that a daily dose of 40 mg would be safe and would deliver complete inhibition of VAP-1.

The VIDI study (VAP-1 Inhibition in DME) was a phase 2 study designed to evaluate ASP8232 safety and effect on excess retinal thickness when given alone or in combination with the anti-VEGF agent, ranibizumab, in patients with CI-DME.

## Methods

### Study design

The VIDI study was a proof of concept, phase 2a, randomized, placebo capsule and sham-intravitreal injection controlled, double-masked clinical trial (www.clinicaltrials.gov; NCT02302079) conducted at 21 centers in the US from 12 Jan 2015 to 12 Aug 2016.

Institutional Review Board (IRB)/Ethics Committee approval was obtained and the study was conducted in accordance with the Declaration of Helsinki, Good Clinical Practice guidelines, and the Health Insurance Portability and Accountability Act; all patients provided informed consent. An independent academic reading center, the Ocular Imaging Research and Reading Center (OIRRC, Sunnyvale, California), served as the Reading Center for the VIDI study; the OIRRC received, stored, processed, and graded all images from the study.

The study was divided into 3 periods: a screening period of 1–4 weeks; a treatment period of 12 weeks, and a follow-up period of 12 weeks. Patients were randomized (1:1:1) to one of three treatment groups: (1) ASP8232 monotherapy: oral ASP8232 40 mg once daily + sham intravitreal injections once per month; (2) combination therapy of ASP8232 and ranibizumab: oral ASP8232 40 mg once daily + ranibizumab (0.3 mg) intravitreal injections once per month; and (3) ranibizumab monotherapy: oral ASP8232-matched placebo once daily + ranibizumab (0.3 mg) intravitreal injections once per month.

During the treatment period, study participants received ASP8232 (40 mg) or placebo once daily from Day 1–84 and an intravitreal injection of ranibizumab or sham on Days 1, 29, and 57. The dose of 40 mg ASP8232 was selected based on previous studies that showed that this dose was safe and was predicted to achieve maximal VAP-1 inhibition over a 24-h period with once daily dosing. All study participants and site staff were masked to all treatments, except for the injecting ophthalmologist, who was not involved in any other study assessments at the study site.

### Participants

Patients were eligible if they were aged 18–85 years with type 1 or type 2 diabetes mellitus with the following specific characteristics: glycated hemoglobin (HbA1c) ≤ 12%; central subfield thickness (CST) ≥ 375 µm in the study eye; early treatment diabetic retinopathy study (ETDRS) BCVA letter score ≤ 73 (Snellen 20/40) and ≥ 24 (Snellen 20/320) in the study eye. Patients with any of the following characteristics were excluded from study participation: macular edema or decrease in BCVA due to a cause other than DME; presence of any other ocular disease in the study eye that may have caused substantial reduction in BCVA; significant macular ischemia or other retinal inflammatory or active periocular or ocular infection; history of noninfectious uveitis, high myopia (− 8 diopter or more correction), pars plana vitrectomy, any ocular surgery, YAG (yttrium–aluminum-garnet) capsulotomy, panretinal scatter photocoagulation, or focal laser within 3 months of study enrollment; history of intravitreal, subtenon, or periocular, non-sustained release, steroid therapy within 3 months before the study; history of intravitreal sustained release dexamethasone therapy within 6 months of study enrollment; history of intravitreal sustained release fluocinolone within 3 years of study enrollment or history of prior treatment with intravitreal VEGF treatment within 8 weeks of study enrollment.

### Study drug

ASP8232 was provided as 40 mg capsules and ASP8232-matched placebo was provided as matching capsules of microcrystalline cellulose. The capsules were taken in the morning with or without food. Ranibizumab was administered as intravitreal injections of 0.05 mL of a 6 mg/mL ranibizumab aqueous solution containing 10 mM histidine HCl, 10% α,α-trehalose dihydrate, 0.01% polysorbate 20 with pH 5.5. Sham intravitreal for ranibizumab was supplied as an empty vial. An anesthetic was administered in the study eye before each intravitreal injection (including the sham intravitreal injection). The sham injection consisted of the (unmasked) injecting ophthalmologist pressing an empty syringe against the surface of the eye to mimic an intravitreal injection. Ranibizumab was injected intravitreally according to the instructions of the manufacturer. Rescue therapy could be considered, under the discretion of the evaluating investigator, if a patient experienced a decrease in ETDRS-BCVA of ≥ 15 letters from baseline or a decrease in ETDRS-BCVA of ≥ 10 letters *and* an increase in CST of ≥ 75 µm. Rescue therapy in the study eye could only be considered after completion of the assessments at Week 4 and was managed by the injecting ophthalmologist. Patients who received rescue therapy with laser photocoagulation were permitted to continue the study; patients who required any other rescue therapy were discontinued from the study treatment. The randomization list and study medication mask were generated and maintained by an interactive website response system.

### Assessments

Both spectral domain-optical coherence tomography (SD-OCT) and ETDRS-BCVA were evaluated at screening, at all visits (Days 1, 15, 29, 57, 85) during the treatment period, during follow-up (Day 113), and at the end of study visit (Day 169). CST was assessed by the Heidelberg Spectralis (Heidelberg Engineering, Heidelberg, Germany) SD-OCT machine. BCVA was assessed according to the ETDRS protocol and all assessments were performed by trained assessors. Fluorescein angiography (FA) and fundus photography were conducted at screening, and on Days 85 and 169. All FA, fundus photography, and SD-OCT images were sent to an independent academic centralized reading center, the OIRRC, for grading and analyses conducted by masked graders. Samples for the assessment of ASP8232 concentrations in plasma were collected during visits on Days 15, 29, 57 prior to oral dosing (approximately 24 h after the previous day dose), and on Days 85, 113, and 169 (approximately 24 h, 4 weeks, and 12 weeks post-dose). Samples for the assessment of ASP8232 concentrations in aqueous humor from the anterior chamber of the study eye were collected on Days 1, 28, and 85. Concentrations of ASP8232 were measured using a validated liquid chromatography-tandem mass spectrometry method. Samples for VAP-1 concentrations and VAP-1 activity were collected during each visit.

### Outcome parameters

The primary efficacy endpoint was the percent change from baseline (%CFB) to Week 12 [end of treatment (EoT)] in excess CST in the study eye, as assessed by SD-OCT. Excess CST was defined as the difference between measured CST and the predefined “normal” value of 320 μm [26, 27]. Secondary endpoints included the absolute change from baseline in CST in the study eye at each visit, and the change from baseline in ETDRS-BCVA score in the study eye at each visit. Pharmacokinetic and pharmacodynamic end points included ASP8232 concentrations in plasma and anterior chamber aqueous humor and VAP-1 concentration and activity in plasma and in aqueous humor. Safety and tolerability of ASP8232 was assessed by monitoring the nature, frequency, and severity of systemic and ocular treatment-emergent adverse events (TEAEs), as well as vital signs, clinical laboratory assessments, and electrocardiograms (ECGs).

### Statistical methods

The VIDI study was designed to reject that the %CFB in excess CST is ≤ 10%, assuming a true %CFB in excess CST of 40% with a standard deviation of 65%. Assuming a drop-out rate of 15%, the total number of patients to be randomized to have an 80% power was planned to be 84 (28 per group). The efficacy analyses were conducted using the full analysis set (FAS), which included all randomised participants who received at least one dose of the study drug and had an efficacy measurement at baseline and at least one at post baseline. The safety analysis set included all participants who received at least one dose of study drug and was used for the demographics and baseline characteristics and also the safety analyses. The pharmacokinetic analysis set included participants from the safety analysis set population for whom sufficient plasma concentration data were collected (i.e. at least one plasma concentration with a recorded dosing time prior to the sample collection). All data analyses were conducted using Statistical Analysis Software^®^ version 9.3 on UNIX.

Efficacy analysis was performed on %CFB in excess CST and change from baseline in ETDRS-BCVA score at 12 weeks. The hypothesis test on the %CFB in excess CST was based on the upper bound of a 2-sided 80% confidence interval (CI) constructed using the t-distribution. If this upper bound was lower than − 10%, then the null hypothesis of no significant effect was rejected, and the correspondent alternative hypothesis was accepted. In addition, a secondary analysis was performed using a mixed model for repeated measures including treatment group, visit and treatment group by visit interaction, as fixed class factors, and baseline excess CST as continuous covariate. Observations after treatment discontinuation were excluded from the primary analysis but reported separately as part of the post-treatment follow-up period.

## Results

### Patient disposition and baseline characteristics

All study patients were consecutively screened and randomized across the participating sites. Of 240 patients who signed informed consent, 96 were randomized to receive ASP8232 monotherapy (N = 32), ASP8232/ranibizumab combination therapy (N = 33), or ranibizumab monotherapy (N = 31). A total of 29 patients (90.6%) in the ASP8232 group, 31 patients (93.9%) in the ASP8232/ranibizumab group and 27 patients (87.1%) in the ranibizumab group completed the study. All 96 randomized patients were included in the safety analysis set; 95 patients were included in the FAS (ASP8232 group, n = 32; ASP8232/ranibizumab group, n = 32; ranibizumab group, n = 31). Table 1 and Additional file 1: Table S1 summarize the patient demographics and baseline characteristics.

**Table 1:** Demographics and baseline characteristics (safety analysis set)

Parameter	ASP8232 (n = 32)	ASP8232/ranibizumab (n = 33)	Ranibizumab (n = 31)	Total (n = 96)
*Sex*				
Male	17 (53.1)	15 (45.5)	16 (51.6)	48 (50.0)
Female	15 (46.9)	18 (54.5)	15 (48.4)	48 (50.0)
*Ethnicity*				
Not Hispanic or Latino	21 (65.6)	18 (54.5)	24 (77.4)	63 (65.6)
Hispanic or Latino	11 (34.4)	15 (45.5)	7 (22.6)	33 (34.4)
*Race*				
White/Caucasian	26 (81.3)	27 (81.8)	22 (71.0)	75 (78.1)
Black/African American	4 (12.5)	5 (15.2)	5 (16.1)	14 (14.6)
Asian	1 (3.1)	0	1 (3.2)	2 (2.1)
American Indian/Alaskan native	0	0	2 (6.5)	2 (2.1)
Other	1 (3.1)	1 (3.0)	1 (3.2)	3 (3.1)
*Age, y*				
Mean (SD)	61.5 (8.1)	59.8 (9.2)	63.4 (8.6)	61.5 (8.7)
Median	61.5	60.0	65.0	62.0
Range	47–82	30–81	45–78	30–82
*Age group (years)*				
≤ 64	22 (68.8)	22 (66.7)	14 (45.2)	58 (60.4)
≥ 65	10 (31.3)	11 (33.3)	17 (54.8)	38 (39.6)
*EudraCT age category (years)*				
≥ 18 to ≤ 64	22 (68.8)	22 (66.7)	14 (45.2)	58 (60.4)
≥ 65 to ≤ 84	10 (31.3)	11 (33.3)	17 (54.8)	38 (39.6)
≥ 85	0	0	0	0
*Weight, kg*				
Mean (SD)	87.66 (19.79)	92.68 (25.33)	92.42 (20.37)	90.92 (21.91)
Median	86.85	83.60	88.20	86.40
Range	53.2–139.5	61.4–157.7	55.9–140.9	53.2–157.7
*Height, cm*				
Mean (SD)	165.74 (9.13)	167.53 (11.31)	167.58 (8.78)	166.95 (9.76)
Median	166.82	167.64	165.10	165.55
Range	146.05–182.88	149.86–190.50	152.40–187.96	146.05–190.50
*BMI, kg/m* ^*2*^				
Mean (SD)	31.92 (6.99)	32.70 (6.80)	33.06 (7.76)	32.56 (7.13)
Median	29.40	30.50	31.10	30.60
Range	21.5–51.1	24.5–54.5	21.8–53.3	21.5–54.5
*Study eye*				
Left	12 (37.5)	20 (60.6)	14 (45.2)	46 (47.9)
Right	20 (62.5)	13 (39.4)	17 (54.8)	50 (52.1)
*Number of qualified eyes*				
1	25 (78.1)	30 (90.9)	27 (87.1)	82 (85.4)
2	7 (21.9)	3 (9.1)	4 (12.9)	14 (14.6)
*CST, study eye (µm)*				
Mean (SD)	535.8 (117.9)	508.2 (104.3)	501.6 (105.5)	515.3 (109.3)
Median	522.0	489.0	488	490.0
Range	367–809	356–827	348–824	348–827
*CST stratification (µm)*				
≤ 500	16 (50.0)	18 (54.5)	16 (51.6)	50 (52.1)
> 500	16 (50.0)	15 (45.5)	15 (48.4)	46 (47.9)
*ETDRS-BCVA, study eye (letters)*				
Mean (SD)	59 (10.1)	59.9 (10.8)	57.7 (14.7)	58.9 (11.9)
Median	61.5	63.0	64.0	63.0
Range	34–78	32–73	9–75	9–78
*Iris color*				
Blue	7 (21.9)	5 (15.2)	6 (19.4)	18 (18.8)
Green	1 (3.1)	1 (3.0)	1 (3.2)	3 (3.1)
Brown	20 (62.5)	25 (75.8)	17 (54.8)	62 (64.6)
Hazel	4 (12.5)	2 (6.1)	6 (19.4)	12 (12.5)
Other	0	0	1 (3.2)	1 (1.0)
*Smoking history*				
Never	22 (68.8)	20 (60.6)	20 (64.5)	62 (64.6)
Current	2 (6.3)	1 (3.0)	2 (6.5)	5 (5.2)
Former	8 (25.0)	12 (36.4)	9 (29.0)	29 (30.2)
*Smoking duration, y*				
n	10	13	11	34
Mean (SD)	21.8 (15.8)	20.5 (11.1)	18.5 (11.8)	20.2 (12.5)
Median	29.5	20.0	20.0	20.0
Range	0–42	2–35	1–40	0–42
*Diabetic medication*				
Metformin	16 (50.0)	15 (45.5)	16 (51.6)	47 (49.0)
DPP-4 inhibitors	5 (15.6)	6 (18.2)	2 (6.5)	13 (13.5)
Insulin for inhalation	1 (3.1)	3 (9.1)	6 (19.4)	10 (10.4)
Insulin, fast-acting	11 (34.4)	10 (30.3)	14 (45.2)	35 (36.5)
Insulin, long-acting	15 (46.9)	16 (48.5)	17 (54.8)	48 (50.0)
Insulins/combinations	14 (43.8)	8 (24.2)	15 (48.4)	37 (38.5)
Sulfonylureas	11 (34.4)	10 (30.3)	11 (35.5)	32 (33.3)
*Previous eye medications*				
Study eye	5 (15.6)	5 (15.2)	5 (16.1)	15 (15.6)
Fellow eye	5 (15.6)	6 (18.2)	8 (25.8)	19 (19.8)
*Ocular interventions, study eye*				
Focal laser	7 (21.9)	6 (18.2)	4 (12.9)	17 (17.7)
Panretinal photocoagulation	5 (15.6)	4 (12.1)	5 (16.1)	14 (14.6)
Cataract/Phacoemulsification	4 (12.5)	3 (9.1)	8 (25.8)	15 (15.6)
YAG capsulotomy	1 (3.1)	1 (3.0)	0	2 (2.1)

Data are presented as n (%) unless otherwise noted

*BCVA* best-corrected visual acuity, *BMI* body mass index, *DPP-4* dipeptidyl peptidase 4 inhibitors, *ETDRS* early treatment diabetic retinopathy study

Overall, 15.6% and 18.8% of patients in the safety analysis set received previous and concomitant eye medications to the study eye, respectively. Treatment groups were well-balanced with respect to clinically important medical history, and the median treatment duration was similar across treatment groups and ranged between 84.0–84.5 days. A minority of patients also had a fellow eye enrolled (ASP8232 group, n = 7; ASP8232/ranibizumab group, n = 3; ranibizumab group, n = 4). The mean absolute CST values at baseline in the FAS were 535.8 µm, 511.8 µm, and 501.6 µm in the ASP8232, ASP8232/ranibizumab and ranibizumab groups, respectively. The mean ETDRS-BCVA scores at baseline were 59.0, 59.8 and 57.7, respectively.

### Primary endpoint: percent change from baseline in excess CST

**Fig. 1:**
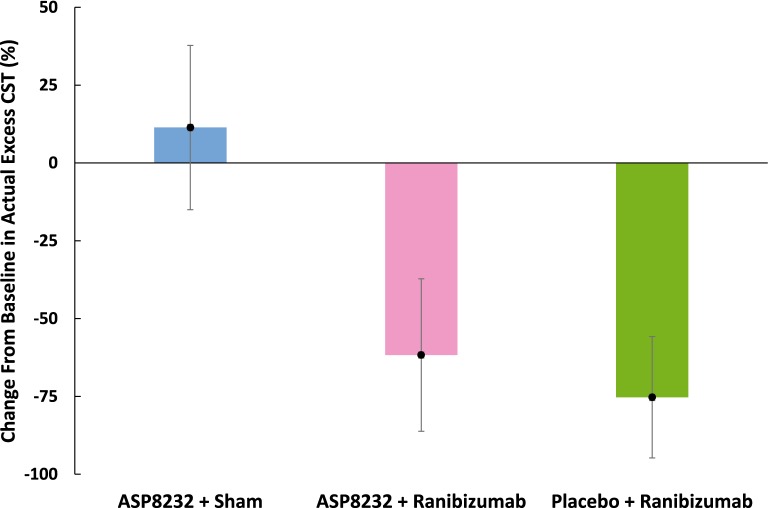
Percent change (95% CI) from baseline in excess CST at EoT visit (FAS). CI, confidence interval; CST, central subfield thickness; EoT, end of treatment; FAS, full analysis set

In the ASP8232 group, the mean %CFB in excess CST to Week 12/EoT in the study eye was 11.4% (95% CI, − 15.0%, 37.8%; *P *= 0.108), and therefore the study did not meet the primary endpoint. The %CFB in the ranibizumab group was − 75.3% (95% CI, − 94.8%, − 55.8%; *P *< 0.001) after 12 weeks. The mean %CFB in excess CST in the study eye in the ASP8232/ranibizumab group showed a somewhat lesser treatment response compared with the ranibizumab group [− 61.7% (95% CI, − 86.1%, − 37.2%); *P *< 0.001] (Fig. 1). ANCOVA analysis of the %CFB in excess CST at Week 12/EoT showed no significant difference between the ASP8232/ranibizumab and the ranibizumab group; however, both groups showed a significant difference compared with the ASP8232 group (*P *< 0.001). In the fellow eye, the %CFB was not statistically significant in any of the 3 treatment groups (data not shown).

### Change in absolute CST

The mean (SD) absolute CST change from baseline was − 123.1(112.3) μm in the ranibizumab group after 12 weeks of treatment, whereas no change was observed in the ASP8232 group. The change from baseline to Week 12 in the ASP8232/ranibizumab group was comparable with the ranibizumab group (Table 2).

**Table 2:** Change in absolute CST values from baseline to Week 12/EoT CIRC-values (FAS)

	ASP8232 (n = 32)	ASP8232/ranibizumab (n = 32)	Ranibizumab (n = 31)
Baseline, μm	535.8 (117.9)	511.8 (103.9)	501.6 (105.5)
Week 12 (EoT), μm	530.3 (127.4)	374.0 (105.6)	378.6 (107.5)
Change from baseline at EoT, μm	− 5.5 (119.7)	− 137.8 (142.5)	− 123.1 (112.3)

Data are presented as mean (SD)

*CIRC* central imaging reading center, *CST* central subfield thickness, *EoT* end of treatment, *FAS* full analysis set

**Fig. 2:**
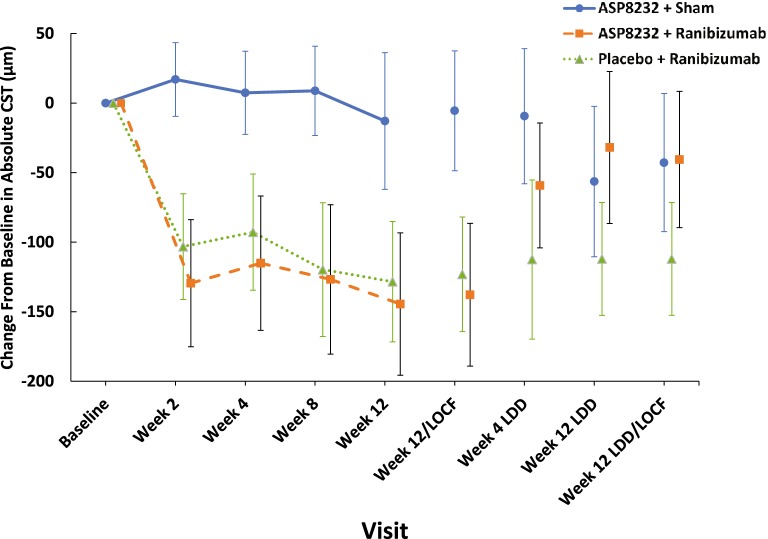
Mean (95% CI) change from baseline in absolute CST values in the study eye (FAS). CI, confidence interval; CST, central subfield thickness; FAS, full analysis set; LDD, last dose date; LOCF, last observation carried forward

CST decreased quickly after the first injection of ranibizumab in the study eye in both ranibizumab and ASP8232/ranibizumab groups, whereas no changes were observed in the ASP8232 treated eyes at any visit. CST values remained stable for up to 12 weeks after the last injection in the ranibizumab group, whereas values tended to return to baseline levels in the ASP8232/ranibizumab group (Fig. 2, Table 3).

**Table 3:** Absolute and percent change from baseline in excess CST to Weeks 2, 4, and 8 (FAS)

	ASP8232	ASP8232/ranibizumab	Ranibizumab
*Baseline*			
n	32	32	31
Mean (SD)	215.8 (117.9)	191.8 (103.9)	181.6 (105.5)
*Week 2*			
n	31	32	30
Mean (SD)	230.9 (134.3)	62.3 (109.1)	71.5 (85.3)
%CFB	16.3 (39.4)	− 69.3 (55.4)	− 57.6 (51.9)
*Week 4*			
n	32	32	31
Mean (SD)	223.1 (130.6)	76.7 (115.0)	88.8 (109.0)
%CFB	11.6 (44.1)	− 56 (57.0)	− 50.2 (64.4)
*Week 8*			
n	32	32	29
Mean (SD)	224.5 (110.1)	64.9 (114.8)	69.4 (118.8)
%CFB	17.7 (57.1)	− 55.1 (77.3)	− 66.0 (57.0)

*CST* central subfield thickness, *FAS* full analysis set, *SD* standard deviation

**Fig. 3:**
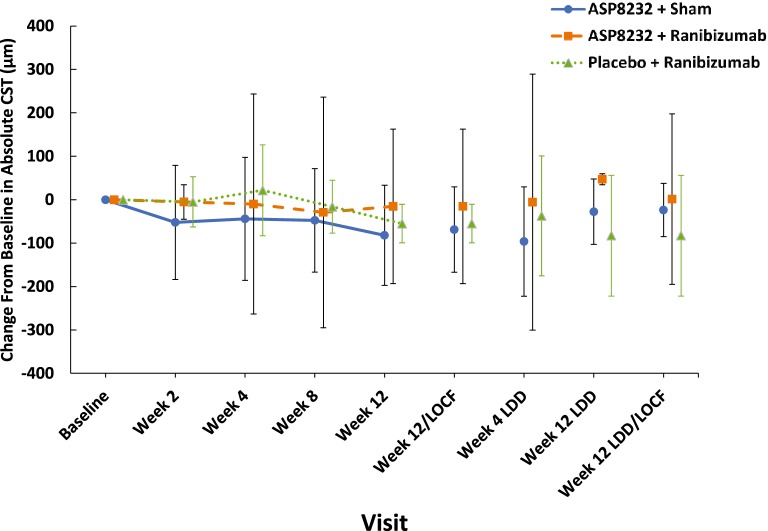
Mean (95% CI) change from baseline in absolute CST values in the fellow eye (FAS). CI, confidence interval; CST, central subfield thickness; FAS, full analysis set; LDD, last dose date; LOCF, last observation carried forward

The mean change from baseline in absolute CST values in the qualified fellow eye at Week 12/EoT was − 82 μm, − 15.3 μm and − 55 μm in the ASP8232, ASP8232/ranibizumab, and ranibizumab groups, respectively (Fig. 3).

### Effect on ETDRS-BCVA

**Fig. 4:**
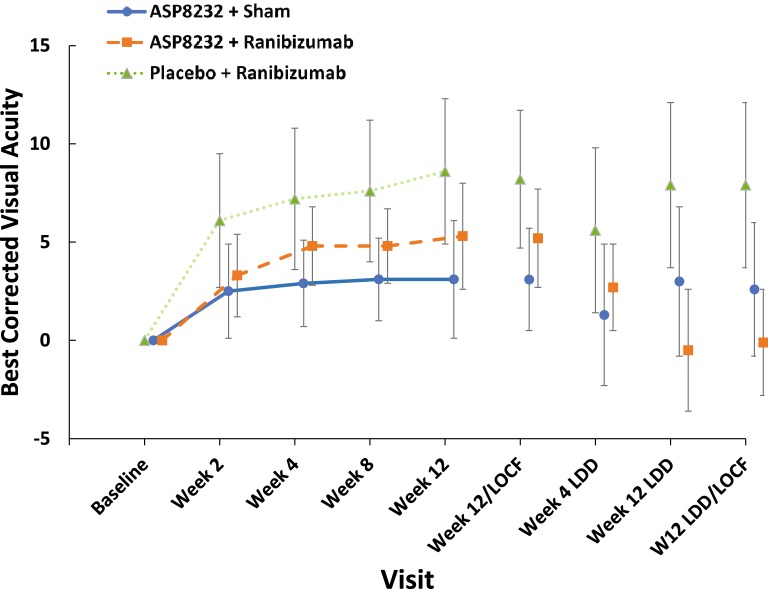
Mean (95% CI) change from baseline in ETDRS calculated BCVA score (FAS). FAS, full analysis set; LDD, last dose date; LOCF, last observation carried forward

The mean (SD) change from baseline in ETDRS-BCVA score to Week 12/EoT was 3.1 (7.3) in the ASP8232 group, 5.2 (7.1) in the ASP8232/ranibizumab group, and 8.2 (9.5) in the ranibizumab group. The change in ETDRS-BCVA score was significantly higher in the ranibizumab group than in the ASP8232 group (*P* = 0.015) (Fig. 4). In the ranibizumab treated eyes, ETDRS-BCVA showed an increase by Week 2 and then continued to improve until week 12/EoT. In the ASP8232 group a small increase in ETDRS-BCVA was observed at week 2, and then ETDRS-BCVA remained at the same level in the ASP8232 group. During the follow-up period, ETDRS-BCVA remained stable until 12 weeks after the last injection in the ranibizumab group and returned to baseline levels in the two remaining treatment groups. At week 2, the number of patients with 5-, 10-, and 15-letter gain in the ETDRS-BCVA was highest in the ranibizumab groups and at week 12, the percentage of patients with ≥ 5-letter gain in ETDRS-BCVA was 48.1%, 64.5%, 69.0% in the ASP8232, ASP8232/ranibizumab, and ranibizumab group, respectively.

### Pharmacokinetic and pharmacodynamic end points

**Fig. 5:**
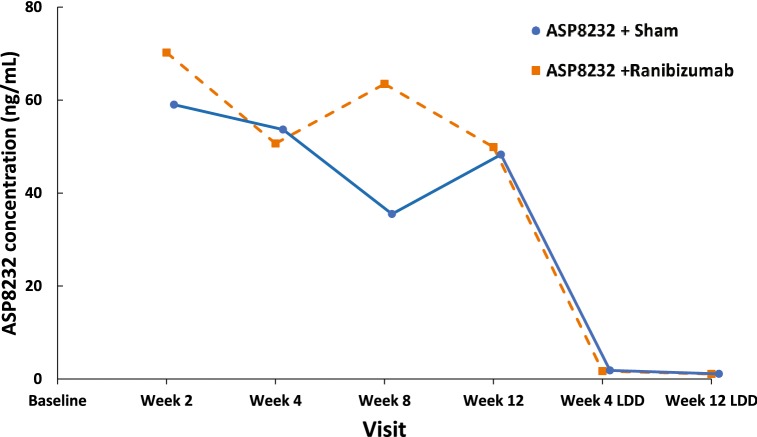
Mean plasma concentration of ASP8232 (pharmacokinetic analysis set). LDD, last dose date

**Fig. 6:**
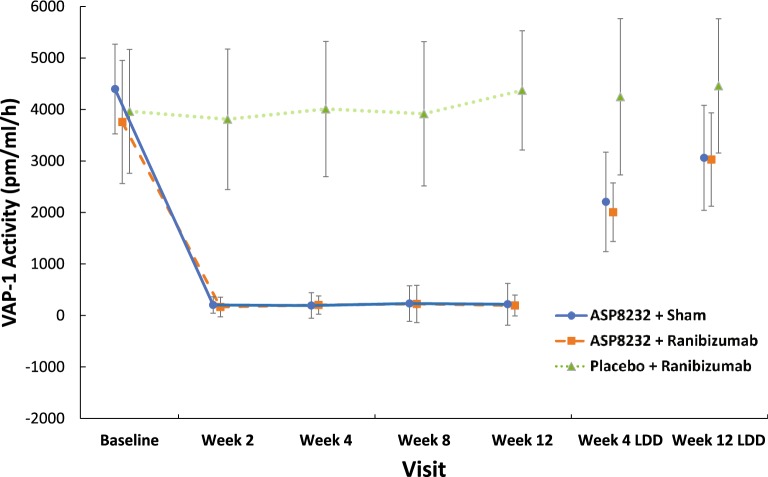
Mean (SD) VAP-1 activity in plasma (pharmacodynamic analysis set). LDD, last dose date

Median trough plasma levels of ASP8232 ranged from 24.1 to 43.10 ng/mL during the treatment period. ASP8232 was still measurable in plasma 4 and 12 weeks after the last dose (Fig. 5). The median ASP8232 levels in the aqueous humor at 12 weeks was 2.98 ng/ml ranging from 0 to 31.1 ng/ml. The median plasma VAP-1 protein levels did not change throughout the study across treatment groups and ranged from 443 to 569 ng/ml. The median VAP-1 protein levels in aqueous humor did not change significantly from baseline to week 12 in any of the 3 treatment groups and ranged from 0.72 to 1.21 ng/ml across treatment groups and time points. In the ASP8232 groups, plasma VAP-1 activity was nearly completely inhibited throughout the treatment period, with median inhibition from baseline to week 12 ranging from 96.8% in the ASP8232/ranibizumab group to 97.3% in the ASP8232 group, indicating that ASP8232 fully inhibits VAP-1 with or without ranibizumab. VAP-1 activity was ~ 50% of baseline within 4 weeks after the last dose of ASP8232 and remained between 70% and 85% of the baseline value within 12 weeks after the last dose. Plasma VAP-1 activity did not change in the ranibizumab group (Fig. 6). In the aqueous humor, the median inhibition of VAP-1 activity was 53.2% in the ASP8232 group compared with 35.4% and 27.9% in the ASP8232/ranibizumab and ranibizumab groups, respectively.

### Safety results

The incidence of overall and ocular TEAE was similar across treatment groups, and most were mild-to-moderate in severity (Table 4; Additional file 2: Table S2). The most common TEAEs were conjunctival hemorrhage [ASP8232/ranibizumab, n = 3 (9.1%)], worsening of type 2 diabetes mellitus [ASP8232, n = 2 (6.3%); ASP8232/ranibizumab, n = 3 (9.1%)] and diabetic retinal edema [ASP8232, n = 3 (9.4%); ASP8232/ranibizumab, n = 1 (3.0%); ranibizumab, n = 2 (6.5%)] (Table 4). A small number of patients reported drug-related TEAE [ASP8232, n = 2 (6.3%); ASP8232/ranibizumab, n = 3 (9.1%); and ranibizumab, n = 3 (9.7%)]. Drug-related ocular TEAEs included retinal disorder [ASP8232, n = 1 (3.1%); ASP8232/ranibizumab, n = 1 (3.0)], metamorphopsia [ranibizumab, n = 1 (3.2%)] and photophobia [ranibizumab, n = 1 (3.2%)]. The number of patients reporting serious TEAEs was generally low [ASP8232 n = 3 (9.4%); ASP8232/ranibizumab, n = 1 (3.0%); and ranibizumab, n = 3 (9.7%)] and none of these events were considered to be related to the study drug.

**Table 4:** Overview of TEAEs (safety analysis set)

	ASP8232 (N = 32)		ASP8232/ranibizumab (N = 33)		Ranibizumab (N = 31)	
	n (%)	E	n (%)	E	n (%)	E
Overall TEAEs	21 (65.6)	43	17 (51.5)	50	19 (61.3)	51
Ocular TEAEs	10 (31.3)	14	13 (39.4)	21	12 (38.7)	23
Systemic TEAEs^a^	17 (53.1)	29	13 (39.4)	29	15 (48.4)	28
Drug-related TEAEs	2 (6.3)	6	3 (9.1)	3	3 (9.7)	3
Drug-related ocular TEAEs	1 (3.1)	1	1 (3.0)	1	2 (6.5)	2
Drug-related systemic TEAEs	2 (6.3)	5	2 (6.1)	2	1 (3.2)	1
Serious TEAEs	3 (9.4)	3	1 (3.0)	1	3 (9.7)	3
TEAEs leading to permanent discontinuation of study drug	2 (6.3)	2	1 (3.0)	1	0	0
Drug-related TEAEs leading to permanent discontinuation of study drug	1 (3.1)	1	0	0	0	0
TEAEs reported in ≥ 5% of patients in any treatment group System organ class Preferred term (MedDRA v15.1)						
Endocrine disorders	0		2 (6.1)		0	
Hypothyroidism	0		2 (6.1)		0	
Eye disorders	8 (25.0)		13 (39.4)		12 (38.7)	
Conjunctival hemorrhage	0		3 (9.1)		0	
Diabetic retinal edema	3 (9.4)		1 (3.0)		2 (6.5)	
Retinal aneurysm	0		0		2 (6.5)	
Retinal exudates	2 (6.3)		2 (6.1)		1 (3.2)	
Visual acuity reduced	0		2 (6.1)		1 (3.2)	
Vitreous floaters	1 (3.1)		2 (6.1)		1 (3.2)	
Vitreous hemorrhage	0		2 (6.1)		2 (6.5)	
Gastrointestinal disorders	1 (3.1)		2 (6.1)		1 (3.2)	
Vomiting	0		2 (6.1)		0	
Metabolism and nutrition disorders	3 (9.4)		3 (9.1)		3 (9.7)	
Worsening type 2 DM	2 (6.3)		3 (9.1)		0	

Data are presented as n (%)

*DM* diabetes mellitus, *E* number of events, *TEAEs* treatment-emergent adverse events

^a^Systemic TEAEs include all non-ocular TEAEs

Three patients experienced TEAEs leading to discontinuation of the study drug. In the ASP8232 group, one patient experienced progression of DME that required rescue treatment and was considered possibly drug-related, and another discontinued due to a diagnosis of prostate cancer diagnosed 1 month after randomization, which was considered not related to the study drug. In the ASP8232/ranibizumab group, one patient discontinued due to progression of metastatic malignant melanoma that had been diagnosed 3 years prior to the start of the trial. The patient had been in remission at enrollment and the condition progressed during the study; this TEAE was not considered to be related to the study drug. No clinically significant differences were observed in the change from baseline for clinical laboratory assessments, vital signs, and ECGs between treatment groups, and no deaths occurred during the study. Intraocular pressure was assessed as a safety measure across the treatment arms. Median levels were comparable across treatment groups and time points and ranged from 14 to 17 mmHg with no significant changes (> 10 mmHg after baseline) reported.

## Discussion

VAP-1 is an endothelial adhesion molecule with semicarbazide-sensitive amine oxidase activity that is expressed in the endothelial cells of the retinal vessels [19]. During inflammation, VAP-1 acts with other leukocyte adhesion molecules to recruit inflammatory cells [19]. Since the VAP-1 enzymatic activity results in the production of toxic metabolites including hydrogen peroxide and aldehydes, which are involved in cellular oxidative stress, it has been postulated that increased levels of VAP-1 may contribute to the development of DR.

The VIDI study assessed the clinical efficacy of the specific VAP-1 inhibitor ASP8232 on excess retinal thickness when administered alone or in combination with the anti-VEGF drug ranibizumab in patients with CI-DME. After 12 weeks of treatment, a statistically significant decrease from baseline in mean excess CST of 75.3% was observed in the ranibizumab group whilst no change was observed in the ASP8232 group. The effect in the combination treatment group was numerically less (but not statistically significant) compared with ranibizumab alone and ASP8232 did not provide any additional benefits on CI-DME. Similarly, changes from baseline in absolute CST of 123.1 μm and 137.8 μm were observed in the ranibizumab and ASP8232/ranibizumab group, respectively, whereas no change was observed in the ASP8232 group.

After 12 weeks of treatment, the mean ETDRS-BCVA score in the study eye increased from baseline by 3.1, 5.2, and 8.2 letters in the ASP8232, ASP8232/ranibizumab, and ranibizumab groups, respectively. These data confirm results from previous studies of ranibizumab and demonstrated that treatment with ranibizumab is effective in improving ETDRS-BCVA in DME. ASP8232 alone provided limited improvement in ETDRS-BCVA of only three letters, which is not considered to be of clinical relevance. Interestingly, the outcomes in the combination group were less favorable compared with the ranibizumab group, which confirms that ASP8232 does not provide relevant improvement in ETDRS-BCVA. The minor effects in the qualified fellow eye suggest some effect on CST but because of the very low number of eyes, these data should be interpreted with caution.

In the ASP8232 groups, VAP-1 activity in plasma was greatly inhibited throughout the treatment period and remained below the baseline value within 12 weeks after the last dose, whereas ranibizumab had no effect on VAP-1 activity, as expected. This confirms that the dose of 40 mg ASP8232 once daily was sufficient to completely inhibit VAP-1 in plasma. VAP-1 protein levels and activity in the aqueous humor were much lower than plasma VAP-1 protein levels and activity; the median concentration of ASP8232 in the aqueous humor was only about 11.5% of plasma trough levels.

It is not known whether VAP-1 plays a role in DR at the level of the retinal vasculature or at the vitreo-retinal interface. We measured VAP-1 levels and activity in the anterior chamber as a possible representation of the activity in the vitreous. However, in this study, VAP-1 activity in the aqueous humor was below the detection limits at all points. Therefore, we cannot draw any conclusions regarding its activity.

Although the VIDI Study did not meet its primary endpoint, we have demonstrated that VAP-1 activity can be inhibited with ASP8232. Recently, VAP-1 inhibition demonstrated a significant benefit in diabetic nephropathy, an end-organ diabetic complication of diabetes (manuscript accepted). Therefore, it would not be appropriate to disregard the VAP-1 pathway in diabetic macular edema and/or diabetic retinopathy based solely on the VIDI study results. Further studies evaluating different modes of delivery and/or concentrations of VAP-1 inhibition for eye diseases such as DME and DR may be warranted to further elucidate this potentially important target of pathophysiology.

Other studies evaluating combination treatment have yielded similar results, suggesting that VEGF is the major driver in the pathophysiology of DME [28]. Another VAP-1 inhibitor is currently being investigated in patients with DR in the absence of center-involved macular edema (www.clinicaltrials.gov; NCT03238963). It is conceivable that the presence of significant CI-DME requires inhibition of VEGF, whilst DR could be reduced by VAP-1 inhibition alone.

Our study had several strengths. Patients were randomly assigned to treatments, and all staff at the investigative sites and the sponsor were masked to treatment allocation. A trained and experienced injecting ophthalmologist performed all intravitreal and intravitreal sham injections and had no other involvement in the trial. Standardized and state-of-the-art measures of efficacy and safety were assessed, and study staff were trained before activation of the site. All images were reviewed and assessed by an independent and experienced reading center and formalized reading protocols were utilized. One potential limitation of our study was that the study duration may have been too short to show additional benefit of the combination treatment. Moreover, VAP-1 protein levels and activity were only measured in the aqueous humor and not in the vitreous.

## Conclusions

ASP8232 was not efficacious in reducing CI-DME in patients with DME. ASP8232 was able to inhibit VAP-1 activity; however, addition of ASP8232 to VEGF inhibition did not provide any added benefits in eyes with DME. ASP8232 was well tolerated and no serious unexpected adverse events were reported. VAP-1 inhibition may not impact DR efficacy; future studies investigating this will provide more insight. The proper route of delivery of ASP8232 for ocular diseases should also be reassessed, as local delivery may be more appropriate than systemic administration.

## Additional files

**Additional file 1: Table** **S1.** Baseline characteristics (safety analysis set).

**Additional file 2: Table** **S2.** Ocular TEAEs (safety analysis set).

## Abbreviations

%CFB: percent change from baseline
BCVA: best corrected visual acuity
BMI: body mass index
CI: confidence interval
CI-DME: center-involved diabetic macular edema
CST: central subfield thickness
DME: diabetic macular edema
DPP-4: dipeptidyl peptidase 4 inhibitors
DR: diabetic retinopathy
ECG: electrocardiogram
ETDRS: early treatment diabetic retinopathy study
EOT: end of treatment
FA: fluorescein angiography
FAS: full analysis set
OIRRC: Ocular Imaging Research and Reading Center
PDR: proliferative diabetic retinopathy
SD-OCT: spectral domain-optical coherence tomography
TEAE: treatment-emergent adverse event
VAP-1: vascular adhesion protein-1
VEGF: vascular endothelial growth factor
VIDI: VAP-1 Inhibition in DME
YAG: yttrium–aluminum-garnet
