# Supplementary material for: Primary outcomes of the VIDI study: phase 2, double-masked, randomized, active-controlled study of ASP8232 for diabetic macular edema
Source: Int J Retina Vitreous. 2019 Aug 1;5:28. doi: 10.1186/s40942-019-0178-7 (PMC6670150; doi:10.1186/s40942-019-0178-7)
Supplement: Supplementary file 1 — Additional file 1: Table S1. Baseline characteristics (safety analysis set). [file 40942_2019_178_MOESM1_ESM.pdf]

Additional File 1. Table S1. Baseline Characteristics (Safety Analysis Set)

Parameter	ASP8232 (n=32)	ASP8232/ranibizumab (n=33)	Ranibizumab (n=31)
HbA1c (%)			
Mean (SD)	8.5 (1.8)	8.02 (1.46)	7.97 (1.36)
Median	7.85	7.8	7.75
Range	6.5–12.0	5.6–11.6	6.2–11.6
Cholesterol (mmol/L)			
Mean (SD)	4.990 (1.318)	4.959 (1.681)	4.519 (0.964)
Median	4.694	4.267	4.500
Range	2.79–8.38	3.23–10.71	2.77–6.90
Triglycerides (mmol/L)			
Mean (SD)	2.064 (0.988)	2.582 (3.483)	1.877 (0.801)
Median	1.727	1.648	1.773
Range	0.77–4.72	0.88–20.66	0.78–3.86
Diastolic Blood Pressure (mmHg)#			
Mean (SD)	77.84 (8.28)	75.59 (8.59)	72.63 (6.51)
Median	79.00	77.00	73.00
Range	53.5–89.5	51.5–90.5	58.0–83.5
Systolic Blood Pressure (mmHg)#			
Mean (SD)	137.17 (14.19)	139.89 (13.78)	132.63 (14.51)
Median	137.25	142.00	130.00
Range	113.5–161.0	111.0–158.0	110.0–180.5

#Mean of the last two assessments.
