# Supplementary material for: Primary outcomes of the VIDI study: phase 2, double-masked, randomized, active-controlled study of ASP8232 for diabetic macular edema
Source: Int J Retina Vitreous. 2019 Aug 1;5:28. doi: 10.1186/s40942-019-0178-7 (PMC6670150; doi:10.1186/s40942-019-0178-7)
Supplement: Supplementary file 2 — Additional file 2: Table S2. Ocular TEAEs (safety analysis set). [file 40942_2019_178_MOESM2_ESM.pdf]

Additional File 2. Table S2. Ocular TEAEs (Safety Analysis Set)

	ASP8232 (N=32)	ASP8232/ranibizumab (N=33)	Ranibizumab (N=31)
**Ocular TEAEs**	10 (31.3)	13 (39.4)	12 (38.7)
**Eye disorders**	8 (25.0)	13 (39.4)	12 (38.7)
Cataract	0	0	1 (3.2)
Cataract nuclear	0	1 (3.0)	0
Conjunctival haemorrhage	0	3 (9.1)	0
Diabetic retinal oedema	3 (9.4)	1 (3.0)	2 (6.5)
Diabetic retinopathy	0	0	1 (3.2)
Dry eye	0	1 (3.0)	0
Foreign body sensation in eyes	0	0	1 (3.2)
Iridocyclitis	0	1 (3.0)	0
Iris adhesions	0	1 (3.0)	0
Lipaemia retinalis	0	0	1 (3.2)
Macular cyst	0	0	1 (3.2)
Macular fibrosis	0	0	1 (3.2)
Metamorphopsia	0	0	1 (3.2)
Photophobia	0	0	1 (3.2)
Retinal aneurysm	0	0	2 (6.5)
Retinal disorder	1 (3.1)	1 (3.0)	0
Retinal exudates	2 (6.3)	2 (6.1)	1 (3.2)
Retinal neovascularisation	0	0	1 (3.2)
Retinal vascular disorder	1 (3.1)	0	0
Scotoma	0	1 (3.0)	0
Vision blurred	1 (3.1)	1 (3.0)	1 (3.2)
Visual acuity reduced	0	2 (6.1)	1 (3.2)
Vitreous detachment	1 (3.1)	1 (3.0)	0
Vitreous floaters	1 (3.1)	2 (6.1)	1 (3.2)
Vitreous hemorrhage	0	2 (6.1)	2 (6.5)
**General disorders and administration site conditions**	0	0	1 (3.2)
Injection site pain	0	0	1 (3.2)
**Injury, poisoning, and procedural complication**	2 (6.3)	0	0
Corneal abrasion	1 (3.1)	0	0
Eye operation complication	1 (3.1)	0	0
**Investigations**	1 (3.1)	0	0
Intraocular pressure fluctuation	1 (3.1)	0	0
**Nervous system disorders**	0	1 (3.0)	0
IVth nerve paralysis	0	1 (3.0)	0

Data are presented as n (%).
